# Supplementary material for: DNA Damage Response in Early Breast Cancer: A Phase III Cohort in the Phobos Study
Source: Cancers (Basel). 2024 Jul 23;16(15):2628. doi: 10.3390/cancers16152628 (PMC11311544; doi:10.3390/cancers16152628)
Supplement: Supplementary file 1 [file cancers-16-02628-s001.zip › Supplementary Figures.pdf]

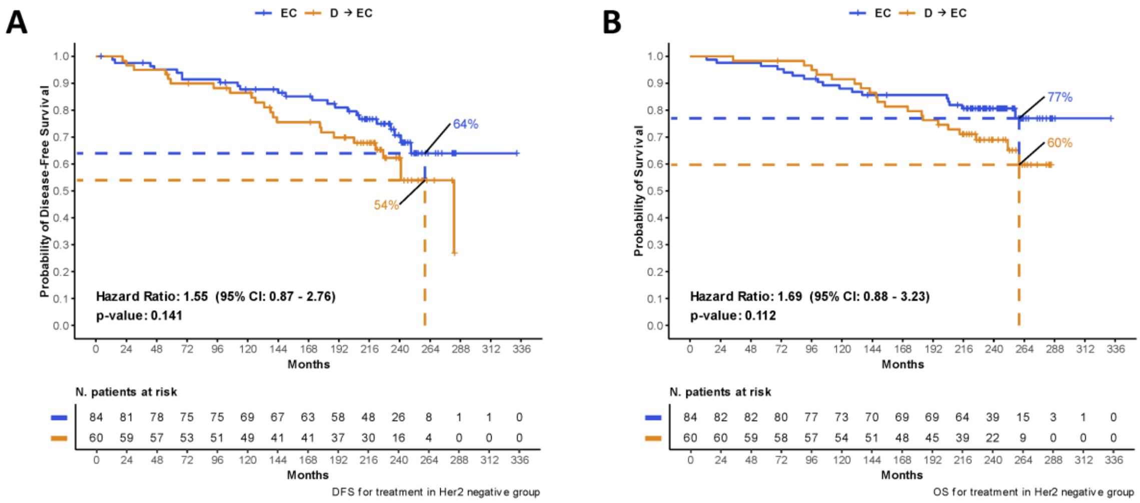

Figure S1. The effect of treatment in the HER2-negative disease subgroup. Panels A and B demonstrate the DFS and OS comparisons, respectively, between EC and D → EC, specifically in patients with HER2-negative disease.

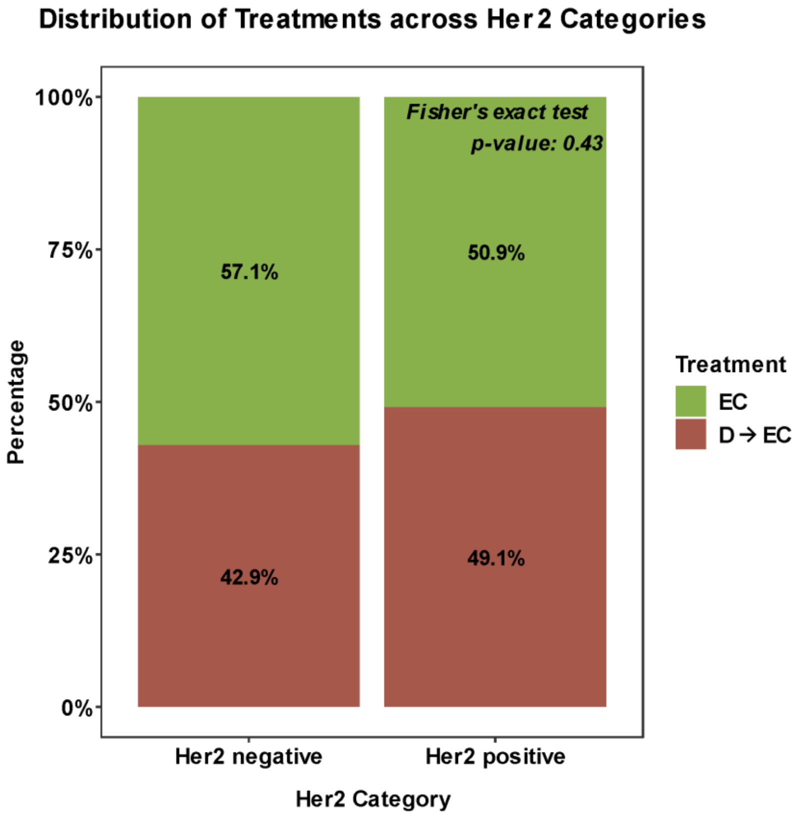

Figure S2. Distribution of treatment arms across classical HER2-subtyping. The stacked bar chart illustrates the distribution of EC and D → EC treatments among patients with HER2-negative (HER2_neg) and HER2-positive (HER2_pos) disease.

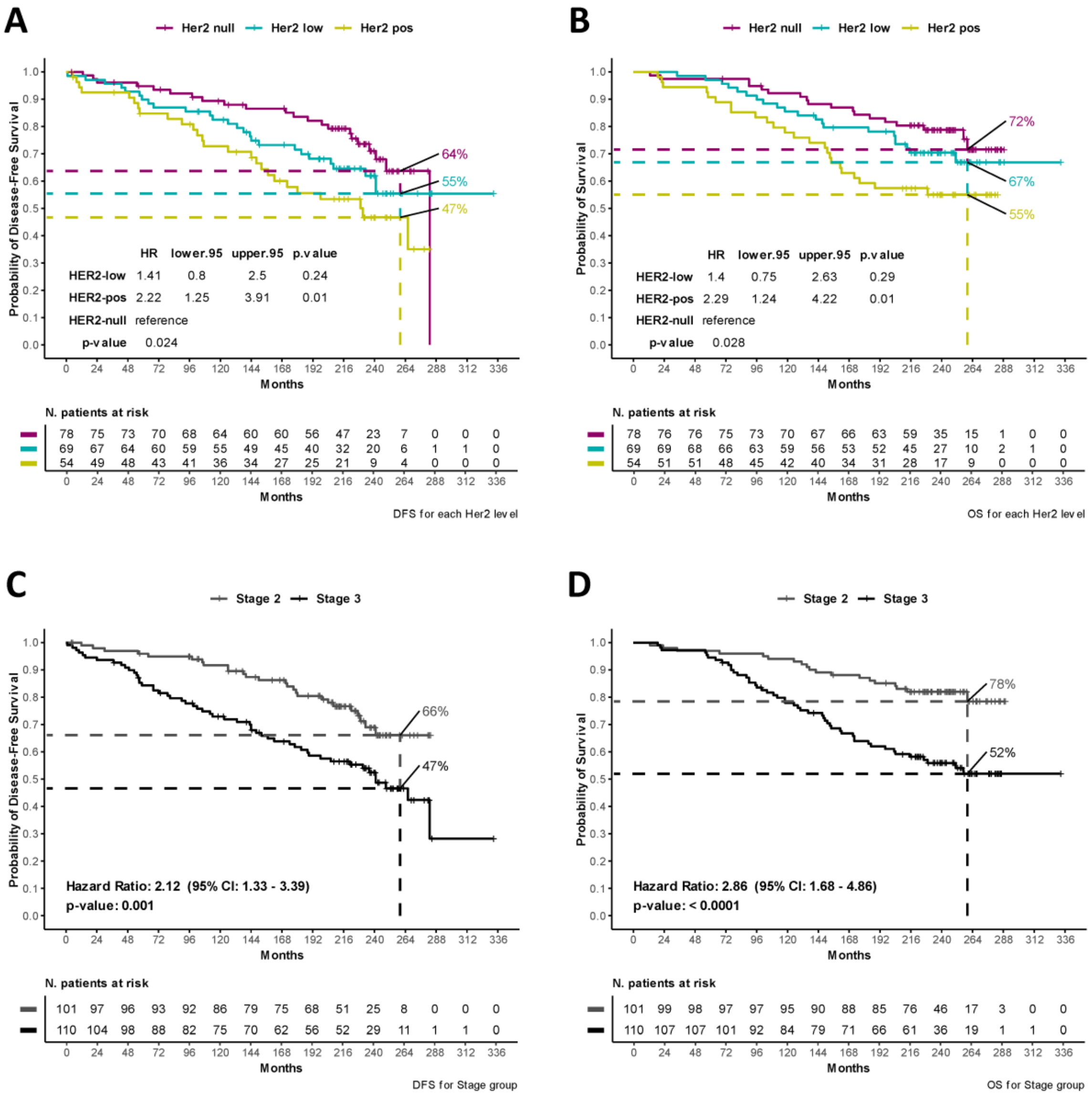

Figure S3. Prognostic impact of HER2 expression levels and stages in terms of DFS and OS. Panels A and B illustrate the KM curves for patients with HER2-positive (HER2_pos), HER2-low, and HER2-null disease, respectively. Panels C and D demonstrate DFS and OS comparisons, respectively, specifically in patients with stage 2 and stage 3 disease.

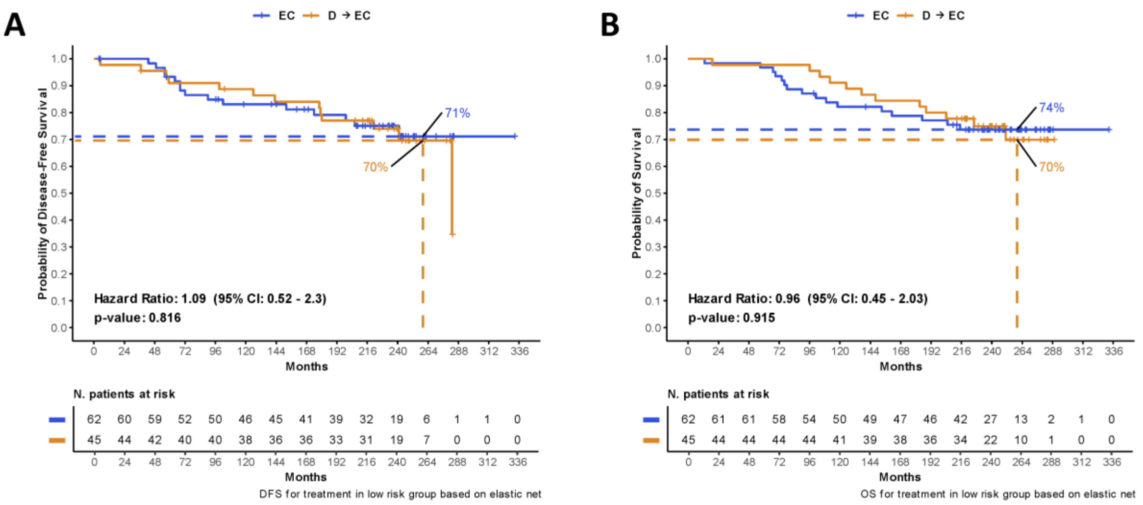

Figure S4. Predictive potential of the DDR risk score. Panels A and B illustrate the KM curves for the DFS and OS comparisons, respectively, between the EC treatment arm and the D → EC treatment arm, specifically in patients with low-risk disease in terms of the DDR risk score.

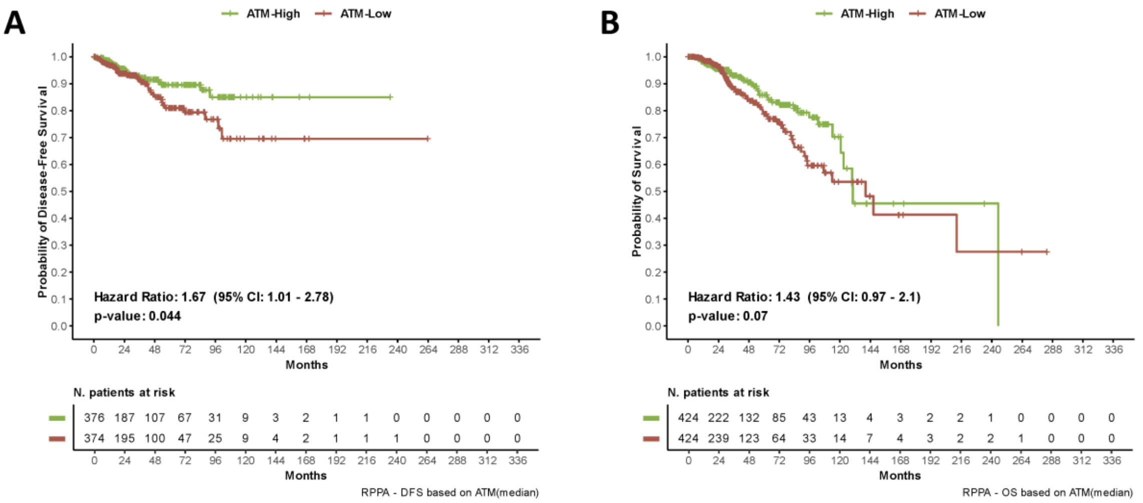

Figure S5. Validation of the ATM prognostic value using RPPA data from the TCGA PanCancer Atlas. Panels A and B illustrate the KM curves for the DFS and OS comparisons, respectively, between patients with ATM-high (higher than the median) and ATM-low (lower than the median) disease.
