# Supplementary material for: DNA Damage Response in Early Breast Cancer: A Phase III Cohort in the Phobos Study
Source: Cancers (Basel). 2024 Jul 23;16(15):2628. doi: 10.3390/cancers16152628 (PMC11311544; doi:10.3390/cancers16152628)
Supplement: Supplementary file 1 [file cancers-16-02628-s001.zip › Table S1.pdf]

Table S1. DDR biomaker expression in the nucleus (_N) and in the cytoplasm (_C), in terms of categorized immunoreactive score.

Biomarker	Positive, Strong (%)	Positive, Intermediate (%)	Positive, Weak (%)	Negative (%)	Unknown (%)
pATM N	16	42	9	28	5
pATM C	2	9	9	78	2
pATR N	44	33	7	14	2
pATR C	4	12	6	76	2
pCHK1 N	2	10	8	78	2
pCHK1 C	12	30	13	42	2
pH2AX N	4	30	15	49	2
pH2AX C	0	0	3	95	2
RPA32 N	24	34	20	20	2
RPA32 C	1	0	3	94	2
pWEE N	6	35	17	40	2
pWEE C	5	11	8	74	2
