# Supplementary material for: DNA Damage Response in Early Breast Cancer: A Phase III Cohort in the Phobos Study
Source: Cancers (Basel). 2024 Jul 23;16(15):2628. doi: 10.3390/cancers16152628 (PMC11311544; doi:10.3390/cancers16152628)
Supplement: Supplementary file 1 [file cancers-16-02628-s001.zip › Table S2.pdf]

Table S2. Complete list of the first degree and second degree terms of the Cox elastic net model. _N and in the cytoplasm (_C), in terms of categorized immunoreactive score. DDR biomaker expression in the nucleus (_N) and in the cytoplasm (_C), in terms of categorized immunoreactive score; (*) indicates interaction terms.

Coefficient	Biomarker term
-0.08	ATR_C*WEE_N
-0.05	ATM_C*CHK1_N
-0.05	ATR_N*ATR_N
-0.04	ATM_N
-0.04	ATR_N
-0.04	ATM_N*ATM_N
-0.04	ATM_C*ATR_N
-0.04	ATR_C*ATR_C
-0.04	H2AX_C*WEE_N
-0.04	WEE_C*WEE_C
-0.03	RPA32_C
-0.03	ATM_C*H2AX_N
-0.03	CHK1_C*WEE_C
-0.02	ATR_C
-0.02	CHK1_N
-0.02	H2AX_C
-0.02	ATM_N*ATR_N
-0.02	ATM_N*CHK1_C
-0.02	ATM_N*H2AX_C
-0.02	ATM_N*RPA32_N
-0.02	ATM_N*RPA32_C
-0.02	ATM_C*ATM_C
-0.02	ATR_N*ATR_C
-0.02	ATR_C*H2AX_N
-0.02	ATR_C*RPA32_N
-0.02	ATR_C*RPA32_C
-0.02	CHK1_C*H2AX_N
-0.02	CHK1_C*RPA32_C
-0.02	CHK1_C*WEE_N
-0.02	H2AX_N*RPA32_N
-0.02	H2AX_C*H2AX_C
-0.02	H2AX_C*RPA32_N
-0.02	RPA32_C*RPA32_C
-0.02	RPA32_C*WEE_N

-0.01	H2AX_N
-0.01	WEE_C
-0.01	ATM_N*WEE_N
-0.01	ATM_C*RPA32_C
-0.01	ATR_N*CHK1_N
-0.01	ATR_N*CHK1_C
-0.01	ATR_N*H2AX_C
-0.01	CHK1_N*H2AX_N
-0.01	CHK1_N*RPA32_N
-0.01	RPA32_N*WEE_C
-0.01	WEE_N*WEE_N
0	CHK1_C
0	ATR_N*RPA32_N
0	ATR_N*RPA32_C
0	ATR_N*WEE_N
0	CHK1_N*CHK1_C
0	CHK1_N*H2AX_C
0	CHK1_N*RPA32_C
0	CHK1_C*H2AX_C
0	H2AX_N*H2AX_N
0	H2AX_C*RPA32_C
0	RPA32_N*RPA32_C
0	WEE_N*WEE_C
0.01	RPA32_N
0.01	WEE_N
0.01	ATM_N*WEE_C
0.01	ATR_N*H2AX_N
0.01	ATR_C*CHK1_N
0.01	CHK1_N*CHK1_N
0.01	CHK1_C*CHK1_C
0.02	ATM_N*ATM_C
0.02	ATM_N*ATR_C
0.02	ATM_N*CHK1_N
0.02	ATM_C*WEE_N
0.02	CHK1_C*RPA32_N
0.03	ATM_C
0.03	ATM_N*H2AX_N
0.03	ATM_C*ATR_C
0.03	ATM_C*RPA32_N

0.03	ATM_C*WEE_C
0.03	RPA32_N*RPA32_N
0.04	ATR_N*WEE_C
0.04	ATR_C*CHK1_C
0.04	ATR_C*H2AX_C
0.04	ATR_C*WEE_C
0.04	CHK1_N*WEE_N
0.04	H2AX_N*H2AX_C
0.04	H2AX_N*WEE_N
0.04	H2AX_N*WEE_C
0.05	H2AX_C*WEE_C
0.05	RPA32_C*WEE_C
0.06	ATM_C*CHK1_C
0.07	RPA32_N*WEE_N
0.08	ATM_C*H2AX_C
0.09	H2AX_N*RPA32_C
0.11	CHK1_N*WEE_C
